# Thermal Conductivity of Graphene-hBN Superlattice Ribbons

**DOI:** 10.1038/s41598-018-20997-8

**Published:** 2018-02-09

**Authors:** Isaac M. Felix, Luiz Felipe C. Pereira

**Affiliations:** Departamento de Física, Universidade Federal do Rio Grande do Norte, Natal, 59078-970 Brazil

## Abstract

Superlattices are ideal model systems for the realization and understanding of coherent (wave-like) and incoherent (particle-like) phonon thermal transport. Single layer heterostructures of graphene and hexagonal boron nitride have been produced recently with sharp edges and controlled domain sizes. In this study we employ nonequilibrium molecular dynamics simulations to investigate the thermal conductivity of superlattice nanoribbons with equal-sized domains of graphene and hexagonal boron nitride. We analyze the dependence of the conductivity with the domain sizes, and with the total length of the ribbons. We determine that the thermal conductivity reaches a minimum value of 89 W m^−1^K^−1^ for ribbons with a superlattice period of 3.43 nm. The effective phonon mean free path is also determined and shows a minimum value of 32 nm for the same superlattice period. Our results also reveal that a crossover from coherent to incoherent phonon transport is present at room temperature for BNC nanoribbons, as the superlattice period becomes comparable to the phonon coherence length. Analyzing phonon populations relative to the smallest superlattice period, we attribute the minimum thermal conductivity to a reduction in the population of flexural phonons when the superlattice period equals 3.43 nm. The ability to manipulate thermal conductivity using superlattice-based two-dimensional materials, such as graphene-hBN nanoribbons, opens up opportunities for application in future nanostructured thermoelectric devices.

## Introduction

Over the past few decades, heat transport in high-performance nanostructured thermoelectric materials has been controlled primarily by the introduction of atomic-scale impurities, interfaces and defects^1–6^. Such structural changes reduce heat flow by scattering phonons diffusely. A recent approach used for controlling nanoscale heat transport involves phonon wave interference effects, such as the one due to specular reflection and transmission of thermal vibrations at interfaces^7–9^. Superlattices are excellent candidates for this approach because of their atomically flat interfaces, since wave-interference effects depend on the interface conditions. Smoother interfaces lead to greater wave interference effects whereas very rough interfaces scatter phonons diffusely. That is to say, high-quality interfaces favor specular reflection and transmission of phonons^8–10^.

A superlattice corresponds to a periodic or quasi-periodic arrangement of different materials, and can be described by a superlattice period which confers a new translational symmetry to the system, impacting their phonon dispersions and subsequently their thermal transport properties^11^. Superlattices are ideal model systems for the realization and understanding of both coherent (wave-like) and incoherent (particle-like) phonon transport. Coherent phonons are subject to wave interference whereas incoherent phonons are subject to diffuse scattering. A prime example has been experimentally demonstrated in GaAs/AlAs superlattices, where the phase of coherent phonons is preserved across interfaces and they can travel ballistically over long distances^7^. Similarly, it has been experimentally verified in epitaxial perovskite oxide superlattices that there is a crossover from coherent to incoherent phonon transport, which manifests itself as a minimum in lattice thermal conductivity as a function of interface density^8^. In spite of the recent advances, the idea of using periodic structures such as superlattices to control thermal transport by manipulating coherent phonons has been around for a few decades^12^. The existence of a minimum thermal conductivity for a given superlattice period, due to the competition between particle and wave nature of phonons in epitaxial perovskite oxide superlattices is one of the most important and long-standing predictions regarding thermal transport in superlattices^10,13^.

Graphene shows weak Umklapp scattering due to its two-dimensional phonon dispersion relation^14,15^. This feature makes graphene attractive for studying coherent phonon transport in nanostructures, such as graphene-hexagonal boron nitride monolayer superlattices (BNC superlattices). The lattice parameter of the honeycomb structures of graphene and hexagonal boron nitride monolayer (hBN) are nearly the same, enabling the synthesis of superlattices with smooth interfaces^16^, which favors specular scattering of phonons, as discussed above. Graphene is a semi-metal^17^ whereas hBN could be seen as its insulating counterpart^18,19^. Concerning their thermal transport properties, graphene presents the highest thermal conductivity among know materials^20–23^, while hBN’s thermal conductivity is one order of magnitude smaller, but still larger than many bulk semiconductors^19,24^. In both graphene and hBN, at room temperature, phonons are the main heat carriers.

Recently, uniform monolayer graphene-hBN structures have been successfully synthesized via lithography patterning coupled with chemical vapor deposition (CVD)^16,25–27^. It was observed that the formation of BNC structures with zigzag interfaces was preferred over that with armchair interfaces during growth^27^. This approach enables fabrication of large-scale hybrid graphene-hBN heterostructures that are continuous and easily transferable to substrates^16^. It has also been shown that these materials possess unusual physical properties, different from pristine graphene and h-BN^25,28–34^. For instance, both theoretical analysis and experimental results show that the band gap of BNC could be tuned by arranging graphene and hBN domains in various ways^25,28–30^. It has been reported that graphene embedded in hBN with zigzag interfaces always originates semiconducting structures^29^. It was also reported that the Seebeck coefficient of BNC superlattices can be 20 times larger than that of graphene, and that it is highly sensitive to the proportion of hBN in the lattice^35,36^. It has also been found that the thermal conductivity of graphene embedded in hBN depends on hBN concentration and cluster size^37–39^. There are other examples of how the chemical and structural diversities in BNC monolayers affect their thermal transport properties^37–40^.

**Figure 1:**
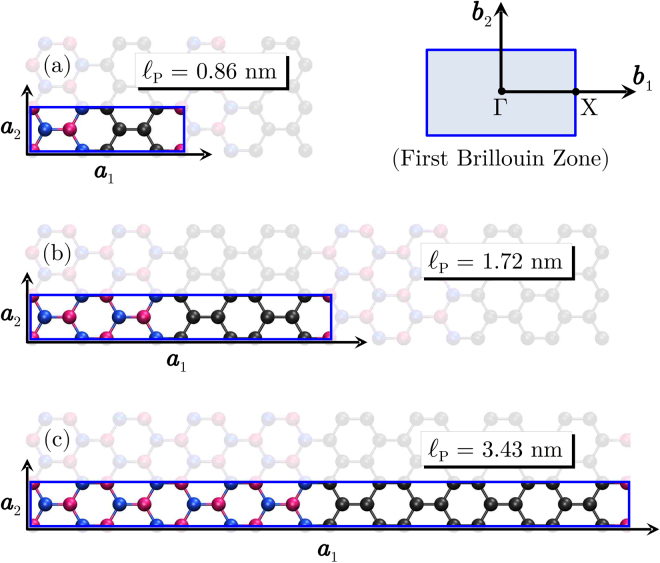
Unit cell of graphene-hBN structures with increasing superlattice period ${\ell }_{{\rm{p}}}$. All ribbons have nominal width of 5 nm and thickness of 0.33 nm. Gray spheres represent carbon atoms, pink spheres are boron atoms and blue spheres are nitrogen atoms. First Brillouin zone and corresponding high-symmetry points are also shown in the top-right panel.

In this work we investigate the heat transport properties of BNC superlattice ribbons with fixed width and equal-sized domains of graphene and hBN, as shown in Fig. 1, via non-equilibrium molecular dynamics simulations. We have considered only zigzag-oriented graphene-hBN interfaces, since those are preferred during growth^27^, but we do not expect our main results to depend on the interface orientation. We analyze the dependence of the conductivity with the domain sizes, and with the total length of the ribbons. We observe a non-monotonic behavior of the conductivity with the superlattice period and identify the corresponding value for which the thermal conductivity is a minimum. Considering the dependence of the conductivity with the length of the ribbons, we determine an effective phonon mean free path (MFP), which also has a minimum value for the same superlattice period.

## Results

### Thermal conductivity dependence with sample length

**Figure 2:**
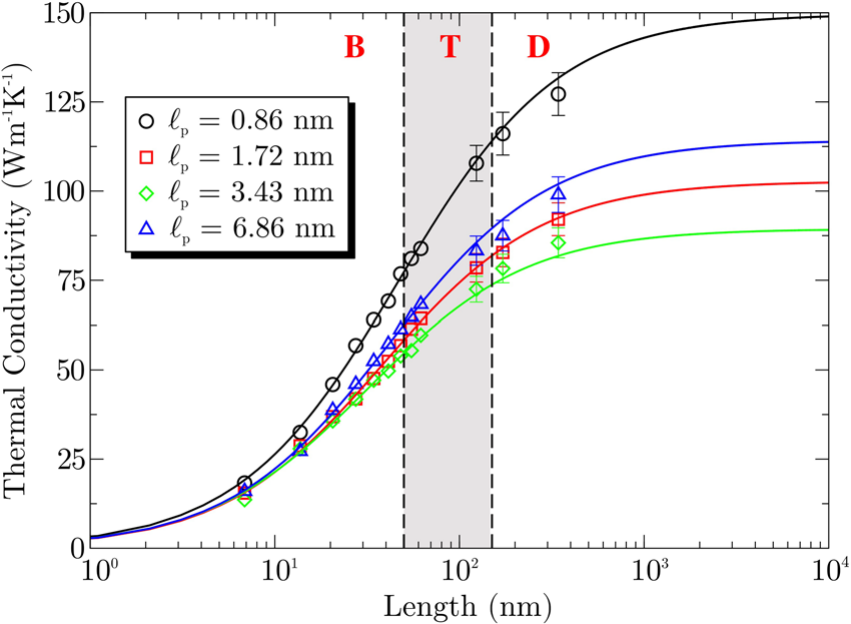
Thermal conductivity as a function of length for increasing superlattice period ${\ell }_{{\rm{p}}}$. The dashed lines indicate the ballistic transport regime (B), the diffusive regime (D), and the ballistic-diffusive transition (T). Lines are fit to Eq. (1), excluding the three largest systems, which shows its predictive power. We only show error bars for larger system sizes, for all others the uncertainties are smaller than symbol sizes.

Due to strong size effects arising from the limitation of the phonon MFP to the region between the heat reservoirs, the conductivity for a system of length *L*_*x*_ is expected to behave as:^41^

1 $$\frac{1}{\kappa ({L}_{x})}=\frac{1}{{\kappa }_{\infty }}(1+\frac{{{\rm{\Lambda }}}_{{\rm{ph}}}}{{L}_{x}}),$$

where *κ*_∞_ is the intrinsic (length-independent) conductivity of the material, and Λ_ph_ is the effective MFP of the heat carriers. Therefore, by fitting the above expression to the simulation data obtained for systems of increasing length we can calculate both, the intrinsic thermal conductivity of the material as well as its effective MFP. In our case, the thermal conductivity is expected to depend on the ribbon length *L*_*x*_, as described in Eq. (1), but also on the superlattice period ${\ell }_{{\rm{p}}}$. From this dependence we can extract the intrinsic thermal conductivity and the effective phonon MFP for each superlattice period. Figure 2 shows the length dependence of *κ* for four superlattice periods, starting from the smallest period considered, 0.86 nm and up to 6.86 nm. For each superlattice period we observe an increase in conductivity with ribbon length, which is described by Eq. (1) represented by the continuous lines in Fig. 2. The fitting was performed without the three longest systems, which were obtained later and present larger uncertainties, but agree with the fitted lines within the error bars. In fact, including the three longer systems in the fitting of Eq. (1), the intrinsic thermal conductivity *κ*_∞_ would change by less than 5%. This agreement shows the remarkable predictive power of Eq. (1), which can be used to predict the intrinsic lattice thermal conductivity from simulations with relatively short systems^42–44^.

Analyzing the behavior of *κ*(*L*_*x*_) we observe three heat transport regimes, as indicated in Fig. 2. First the ballistic regime where *κ* ∝ *L*_*x*_, which is valid in the small *L*_*x*_ region B, up to ≈50 nm. In this region the phonon MFP is larger than the system length, and is thus limited by it. For ${L}_{x}\mathrm{ > 150}$ nm, we observe the diffusive regime where *κ* shows a weak dependence on system length. Here the phonon MFP is shorter than the system length. Finally, between these two regions we find a ballistic-diffusive transition regime, region T, where the system length becomes comparable to the phonon MFP and the dependence of *κ* on *L*_*x*_ decreases.

### Thermal conductivity and effective phonon MFP as a function of superlattice period

**Figure 3:**
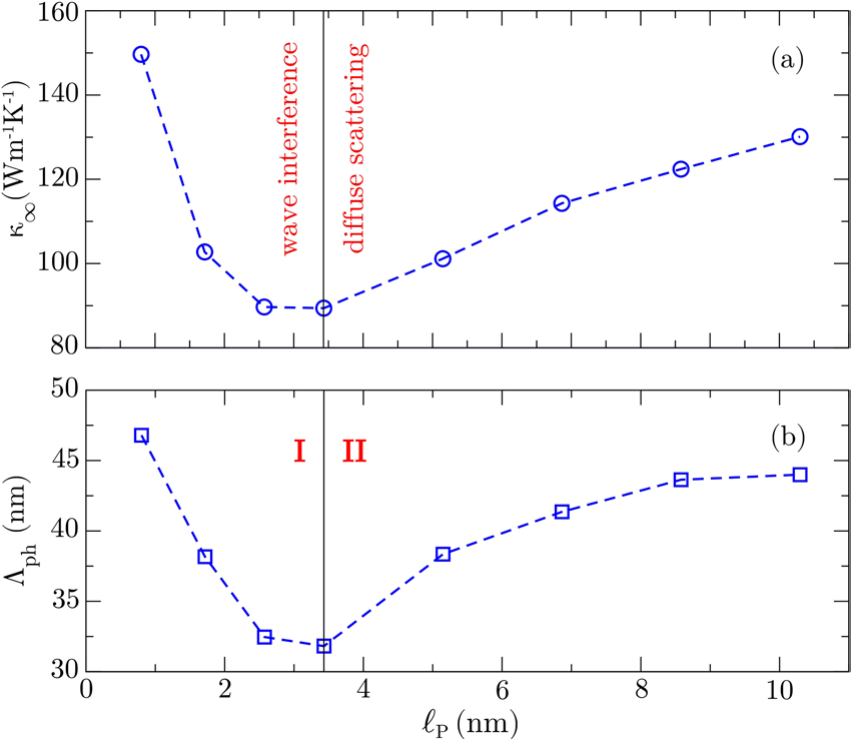
(**a**) Intrinsic thermal conductivity and (**b**) effective phonon mean free path as a function of superlattice period. Both quantities reach a minimum value at ${\ell }_{{\rm{p}}}=3.43$ nm, which marks the interface between coherent and incoherent phonon transport. The dashed lines are just a guide to the eye.

In Fig. 3(a) we present the intrinsic thermal conductivity *κ*_∞_ of the BNC nanoribbons as a function of superlattice period ${\ell }_{{\rm{p}}}$ at 300 K. We observe that the overall superlattice thermal conductivities are remarkably reduced, by ~98% when compared with the thermal conductivity of graphene^20,22,23^, and by ~78% when compared with the thermal conductivity of hBN^24,45^. Another noticeable feature in Fig. 3(a) is the non-monotonic dependence of *κ*_∞_ on ${\ell }_{{\rm{p}}}$. An increase in ${\ell }_{{\rm{p}}}$ initially causes *κ*_∞_ to decrease until it reaches a minimum value of 89 W m^−1^K^−1^ when ${\ell }_{{\rm{p}}}=3.43$ nm, and then it increases. Our results are in general agreement with previous reports which investigated the influence of the superlattice period on the thermal conductivity of BNC superlattices. The observation of a minimum thermal conductivity for a specific superlattice period, as found in our simulations, has been reported by Jiang *et al*.^46^, Zhu and Ertekin^47^, da Silva *et al*^48^. and Chen *et al*.^49^. However, in our work there are two factors not considered in previous works. First, we deal with nanoribbons rather than 2D superlattices, as done in the previous works^46–49^. Therefore, our systems are expected to present more of a 1D character than a 2D one. Second, we consider the intrinsic thermal conductivity of the BNC nanoribbons by employing Eq. (1), while previous works have reported length-dependent conductivities^46–49^. Our numerical estimate for the superlattice period which yields the minimum thermal conductivity is in excellent agreement with the one reported by Chen *et al*.^49^, although their minimum conductivity is approximately twice as much as ours, which can be explained by the fixed system length they used or due to the different width of their supercell.

In general, the minimum thermal conductivity is a consequence of a crossover from coherent to incoherent phonon transport. To the left of the minimum, Brillouin zone folding occurs due to the phonon wave effect, which explains the reduction of thermal conductivity when increasing ${\ell }_{{\rm{p}}}$^49^. To the right of the minimum, the number of interfaces (or thermal resistors) decreases with ${\ell }_{{\rm{p}}}$, thus easing heat conduction^50–53^. Indeed, it has been shown that thermal conductivity decreases when the structure periodicity is dominated by wave interference effects and increases when it depends on diffuse interface scattering^10^. This can also be understood considering that, in general, low-frequency phonons are more likely to experience wave interference effects, such as specular reflection and transmission, due to their large wavelengths, whereas high-frequency phonons are likely to be scattered diffusely at the interfaces^9,10^. Thus, the combination of wave interference effects and diffuse interface scattering leads to a local minimum of thermal conductivity as a function of superlattice period.

Nonetheless, for phonons experiencing wave interference, the thermal conductivity decreases with superlattice period due to the modification of the bulk phonon dispersion relation. This is caused by zone folding and band flattening, which reduce phonon group velocities as the lattice period increases, thereby decreasing the thermal conductivity^54^. Therefore, we can say that, the combination of wave interference effects and diffuse interface scattering leads to a local minimum of thermal conductivity as a function of superlattice period^10^. Note that, for region II in Fig. 3, *κ* rises with increasing period, an indicator that the thermal energy is carried primarily by particle-like phonons (incoherent) that are scattered diffusely at the interfaces. On the other hand, for region I, *κ* decreases with increasing period. This behavior is not compatible with the presence of diffuse scattering only, and one can assume that part of the heat is carried by wave-like phonons (coherent) experiencing interference effects. Thus, the observation of a minimum thermal conductivity as a function of superlattice period presents direct evidence of the crossover from coherent to incoherent phonon transport in these superlattices. Our results reveal that wave interference for thermal phonons and the crossover from coherent to incoherent phonon transport can be present at room temperature for BNC nanoribbons. Similar behavior has been observed in experiments with epitaxial perovskite oxide superlattices^8^.

The lowest thermal conductivity is observed when ${\ell }_{{\rm{p}}}$ is comparable to the phonon coherence length in the superlattice^11,47^. This critical length can be much smaller than the effective phonon MFP of the superlattices. In our simulations, the smallest thermal conductivity *κ*_∞_ = 89 W m^−1^K^−1^ was found for a superlattice with ${\ell }_{{\rm{p}}}=3.43$ nm. Thus, this corresponds to the coherence length of phonons in graphene-hBN superlattice ribbons, in agreement with the predictions by Zhu and Ertekin^47^. From the data in Fig. 3(b) we estimate Λ_ph_ in a superlattice with ${\ell }_{{\rm{p}}}=3.43$ nm to be 32 nm. This value is one order of magnitude larger than the coherence length, also in agreement with Zhu and Ertekin^47^. Notice that phonons experience ballistic transport for system lengths shorter than Λ_ph_, which can reach distances much larger than the coherence length, in agreement with the increasing trend in thermal conductivities shown in Fig. 2.

### Phonon dispersions

In order to better understand the dependence of the thermal conductivity on the superlattice period we have investigated the phonon dispersion for several periods. The phonon dispersion relations were calculated with the General Utility Lattice Program (GULP), which implements lattice dynamics methods^55^. The interatomic potential used was the same as in the MD simulations. Figure 1 illustrates the unit cells with superlattice periods of 0.86 nm, 1.72 nm and 3.43 nm. It also shows the first Brillouin zone of the structures, along with its high-symmetry points. For ${\ell }_{{\rm{p}}}=0.86$ nm, the unit cell is composed of 8 non-equivalent atoms, 4 carbon atoms (gray spheres), 2 boron atoms (pink spheres) and 2 nitrogen atoms (blue spheres). In Fig. 1 we also illustrate the first Brillouin zone for our unit cell, with high-symmetry points Γ = (0, 0, 0), X = ($\tfrac{1}{2}$, 0, 0), in units of reciprocal lattice vectors.

**Figure 4:**
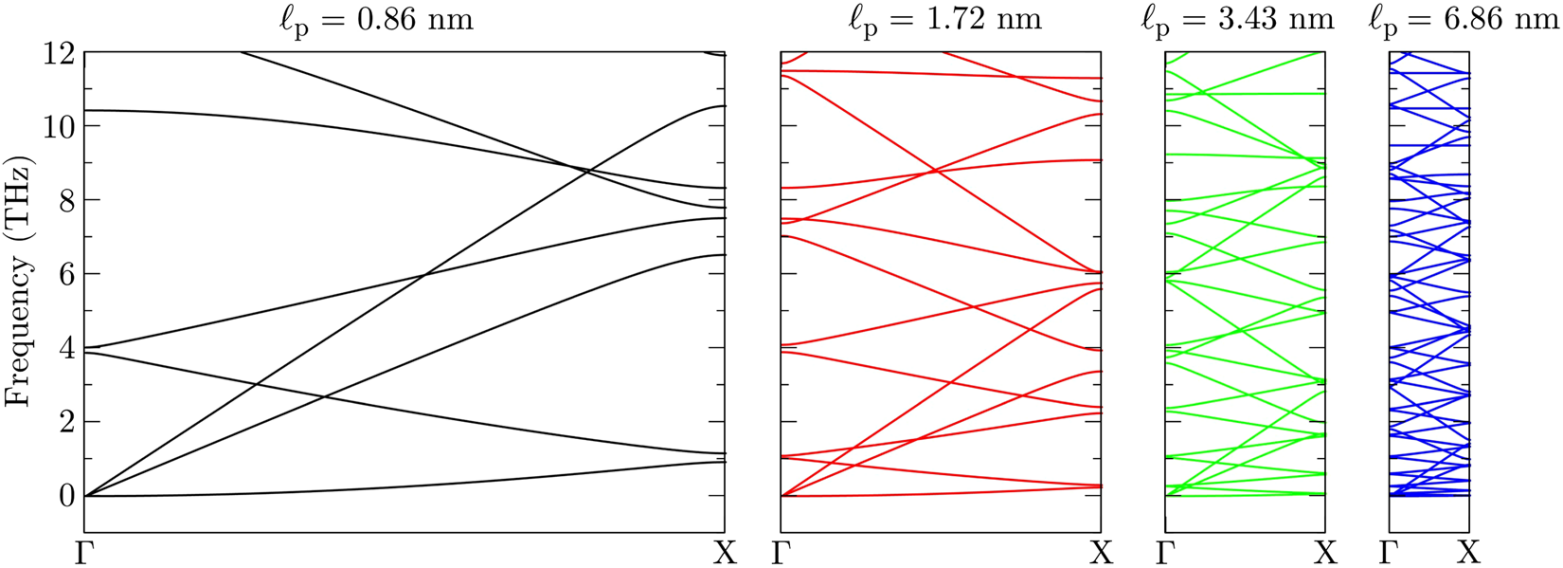
Phonon dispersion for superlattices of increasing period. Only the region around the acoustic phonon modes is shown.

In Fig. 4 we present phonon dispersions for increasing superlattice periods. Only the frequency range covered by the acoustic modes is shown, since those are the main heat carriers. It is important to remember that the number of non-equivalent atoms in the unit cell is proportional to the period, so that the number of vibrational modes follows the same trend. There is a reduction of phonon group velocities (slope of the dispersion curves) with increasing superlattice period due to zone folding. This explains the reduction of thermal conductivity in region I of Fig. 3, where coherent phonons dominate thermal transport and where there must be a superposition of Bloch waves^10,56^. However, this analysis cannot elucidate what happens in region II of Fig. 3, where diffuse scattering dominates, because boundaries scatter phonons diffusely. It is commonly assumed that such scattering processes randomizes the phonon phases such that interference effects, and the resultant modification of the phonon dispersion, can be neglected^7^.

### Vibrational spectrum and period-induced changes in phonon populations

In order to understand the physical origins of the minimum thermal conductivity for a specific superlattice period, we analyze the vibrational spectrum of BNC superlattices. First, we calculated the velocity autocorrelation function (VACF) by post processing 100 ps trajectories, in which atomic velocities are printed out every 5 fs. The VACF is then normalized such that VACF(*t*  = 0) = 1, and averaged over all atoms in the supercell. The vibrational density of states (VDOS) is then calculated from the Fourier transform of the averaged VACF

2 $${\rm{VDOS}}(\omega )={\int }_{0}^{\infty }\frac{\langle v\mathrm{(0)}\cdot v(t)\rangle }{\langle v\mathrm{(0)}\cdot v\mathrm{(0)}\rangle }{e}^{-i\omega t}dt,$$

where *v* is the atomic velocity, 〈*v*(0) ⋅ *v*(*t*)〉 is the VACF and *ω* is the angular frequency.

**Figure 5:**
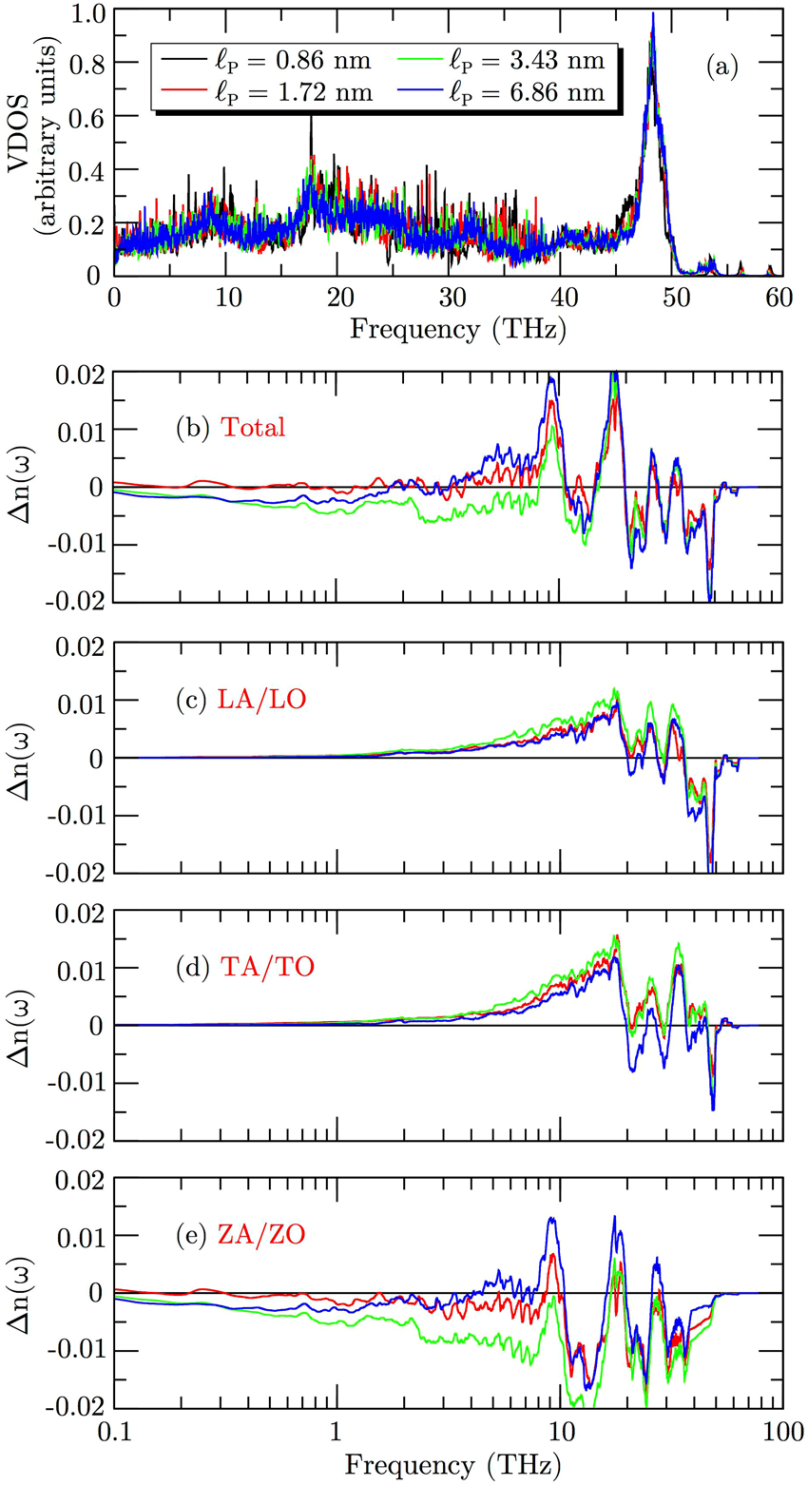
(**a**) Vibrational density of states for BNC superlattices. (**b**–**e**) Changes in phonon populations due to superlattice period increase relative to ${\ell }_{{\rm{p}}}=0.86$ nm.

In Fig. 5(a) we present the total VDOS for each ${\ell }_{{\rm{p}}}$. The number of pronounced peaks decreases as the superlattice period increases, which is due to the increase in size of the unit cell. In general, pronounced peaks in the phonon spectra indicate the presence of coherent phonons. Therefore, as ${\ell }_{{\rm{p}}}$ increases, fewer coherent phonons should be present in the superlattice. For two-dimensional materials, the flexural modes ZA/ZO are the major contributors in thermal transport, while longitudinal LA/LO and transverse TA/TO modes play a smaller role^21,23,57–59^.

Figure 5(b–e) show the changes in phonon populations due to an increase in superlattice period, relative to ${\ell }_{{\rm{p}}}=0.86$ nm, which are calculated from the ratio between the occupation of phonon modes, defined as

3 $${\rm{\Delta }}{\rm{n}}(\omega )=\frac{1+{\int }_{0}^{\omega }{{\rm{VDOS}}}_{{\ell }_{{\rm{p}}}}d\omega ^{\prime} }{1+{\int }_{0}^{\omega }{{\rm{VDOS}}}_{{\ell }_{{\rm{p}}}\mathrm{=0.86}{\rm{nm}}}d\omega ^{\prime} }-1.$$

Therefore, Δn(*ω*) = 0 corresponds to no change in occupation, Δn(*ω*) < 0 indicates a decrease in population and Δn(*ω*) > 0 to an increase relative to ${\ell }_{{\rm{p}}}=0.86$ nm. Notice that for ${\ell }_{{\rm{p}}}=1.72$ nm the total occupation of phonon modes shows no major alterations up to frequencies around 4 THz, while there is a clear decrease in phonon populations in the low-frequency region for superlattice periods ${\ell }_{{\rm{p}}}=3.43$ nm and ${\ell }_{{\rm{p}}}=6.86$ nm. Furthermore, our data shows that the largest decrease in populations happens for ${\ell }_{{\rm{p}}}=3.43$ nm, which is the superlattice period for the structures with the lowest conductivity. Analyzing the changes in populations for each polarization branch, we notice a small increase of LA/LO and TA/TO modes up to frequencies of 20 THz. In panel (e) we notice a pronounced decrease in phonon populations for ZA/ZO modes, and that the largest decrease below 10 THz happens for ${\ell }_{{\rm{p}}}=3.43$ nm. Therefore, we can attribute the minimum thermal conductivity observed for BNC superlattices with a period of 3.43 nm to the reduction in population of flexural phonons for that period. This behavior has not been considered in any of the previous works dealing with BNC superlattices.

## Discussion

**Figure 6:**
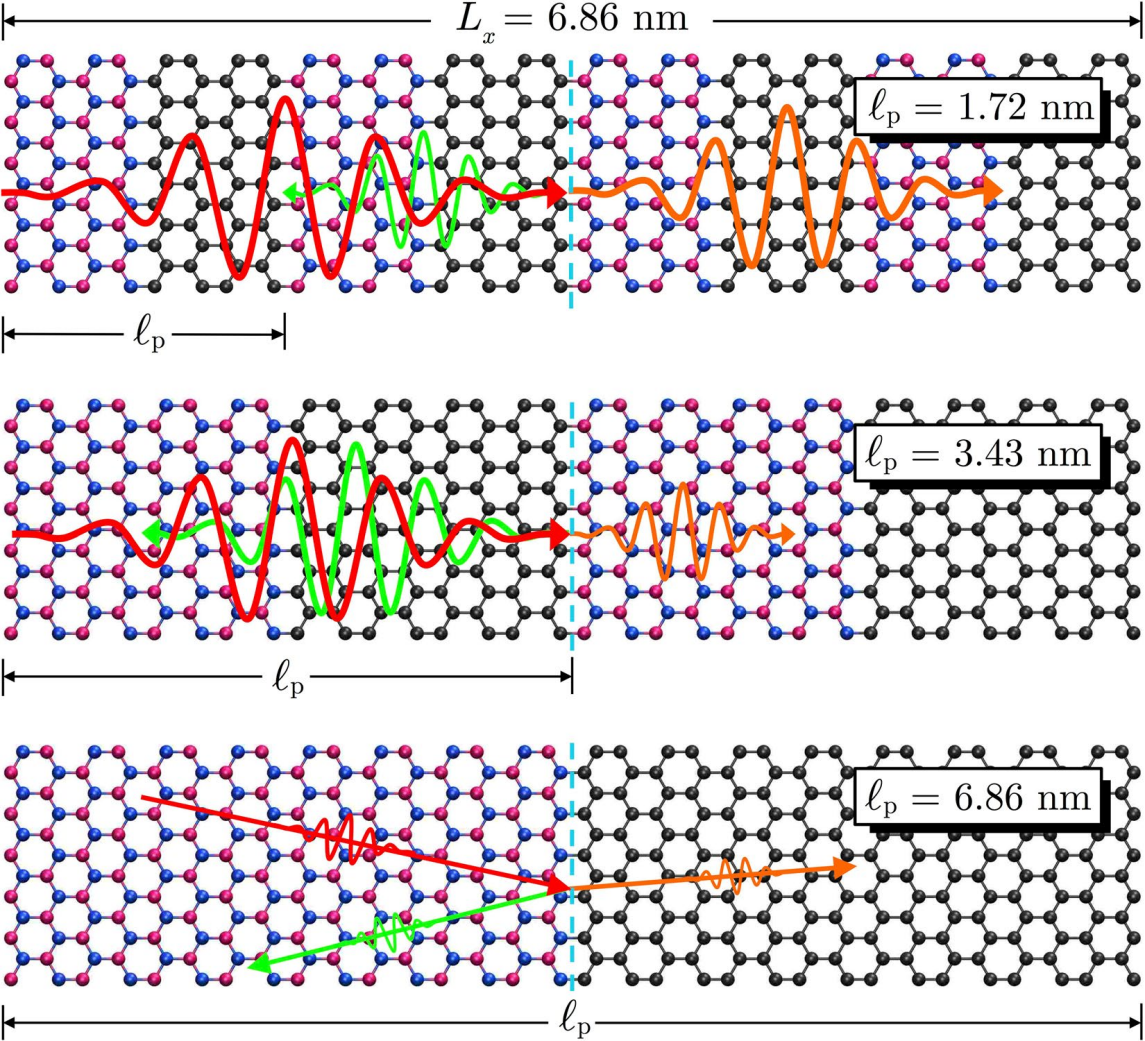
Representative scheme of both coherent (wave interference) and incoherent (diffuse scattering) phonon transport.

From our interpretation of the physical origins of the conductivity reduction in BNC superlattices, we can construct a pictorial representation of phonon scattering at the interfaces between graphene and hBN, which is shown in Fig. 6. For ${\ell }_{{\rm{p}}} < 3.43$ nm, the wavelength of heat carrying phonons is larger than the individual domains, so they suffer small influence of the interfaces, and transport is coherent. For ${\ell }_{{\rm{p}}} > 3.43$ nm, the wavelength of heat carrying phonons is smaller than the individual domains, and they experience a larger influence of the interfaces, therefore transport is incoherent. In the case of ${\ell }_{{\rm{p}}}=3.43$ nm the wavelength of heat carrying phonons is comparable to the size of individual domains, and we have a transition from the coherent to the incoherent transport regimes, which is responsible for the minimum thermal conductivity observed for this superlattice period.

Finally, it is important to comment on the possible experimental realization of these BNC superlattice ribbons. Our results should be seen as an upper bound for the conductivities that could be measured in experiments, since we consider perfect superlattices, with perfect edges and interfaces, and in the absence of isotopic disorder. Any experimental realization of these superlattices is bound to present some of these defects, which are known to reduce heat transport. Another limitation is related to the superlattice period which yields the minimum conductivity. Producing a BNC superlattice with a period smaller than 5 nm could be challenging. Therefore, it is possible that the experiments would not observe the minimum value of *κ*_∞_ but only the weakly increasing trend observed in region II of Fig. 3.

## Methods

Molecular dynamics (MD) simulations in the present work were performed with LAMMPS (Large-scale Atomic/Molecular Massively Parallel Simulator)^60^. We employed the Tersoff empirical potential recently re-parametrized to accurately reproduce the vibrational properties of carbon and hBN nanostructures^39,61,62^. The thermal conductivity of BNC nanoribbons was calculated via non-equilibrium molecular dynamics (NEMD) simulations with periodic boundary conditions along the heat current direction, and free boundary conditions in the other directions. In all simulations the equations of motion were integrated with a 0.5 fs timestep. The systems were initially thermalized with a Nosé-Hoover thermostat at 300 K for 100 ps. Each ribbon was relaxed at finite temperature in order to achieve zero-stress along the periodic direction, the stress along the other two directions is zero on average. The thermostat was turned off once the system reached equilibrium, such that the equations of motion were then integrated under microcanonical conditions.

**Figure 7:**
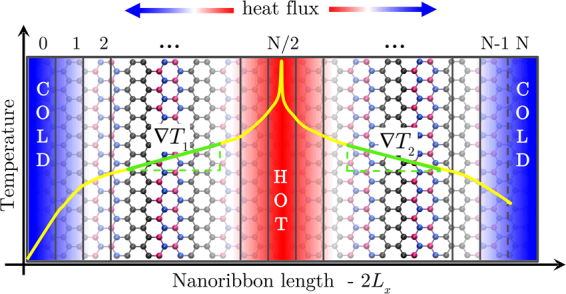
Set-up for the reverse NEMD method. A heat flux is imposed by exchanging the kinetic energy of slow particles in the hot region with fast particles in the cold region. The image of the cold layer becomes the *N*-th layer, such that layers 0 and N are the same. Also shown is the temperature profile from which the temperature gradient is calculated.

We employed the so-called reverse NEMD method, proposed by Müller-Plathe, to impose a heat flux in the system^63^. The heat flux is imposed by exchanging the kinetic energy of slow moving particles in the “hot” region with fast moving particles in the “cold” region, as shown in Fig. 7. The cold region is at the left end of the simulation box, while the hot region is at its center. Due to periodic boundary conditions, the image of the cold region becomes the *N*-th layer, such that regions 0 and N are the same. On average each region had 100–200 atoms in total. The kinetic energy swaps were performed every 1000 timesteps. The heat flux is obtained from the difference in kinetic energy of the exchanged particles as

4 $$J(t)=\frac{1}{2tA}\sum _{exchanges}\frac{{m}_{i}{v}_{i}^{2}-{m}_{j}{v}_{j}^{2}}{2},$$

where *A* is the cross sectional area of the sheet which we define as the width of the ribbon multiplied by its thickness. All ribbons have nominal width of 5 nm (in y-direction) and we assume a thickness of 0.33 nm for graphene and hBN (in z-direction).

After a transient time interval the stationary regime is achieved, where the heat flux reaches a constant average value. In most simulations the stationary regime is stablished after 20 ns, corresponding to 40 × 10^6^ simulation steps. With the system in its stationary regime, we divide it in several slabs along the direction of the heat flux and calculate the temperature in each slab from the average kinetic energy of the particles within the slab, according to the equipartition theorem, as:

5 $${T}_{i}=\frac{2}{3{n}_{i}{k}_{B}}\sum _{j}\frac{{p}_{j}^{2}}{2{m}_{j}},$$

where *T*_*i*_ is the temperature of *i*-th slab, *n*_*i*_ is the number of atoms in *i*-th slab, *k*_*B*_ is Boltzmann’s constant, *m*_*j*_ and *p*_*j*_ are atomic mass and momentum of atom *j*, respectively. Thus, the temperature gradient is calculated from the average temperature in each region of the system.

Once the heat flux and the temperature gradient are stationary we obtain the thermal conductivity for a sample of size *L*_*x*_ directly from Fourier law

6 $$\kappa ({L}_{x})=\frac{\langle {J}_{x}\rangle }{{\nabla }_{x}T},$$

where ∇_*x*_*T* is the arithmetic mean of the temperature gradient considering both directions of heat transport (as shown in Fig. 7)

7 $${\nabla }_{x}T=\frac{|{\nabla }_{x}{T}_{1}|+|{\nabla }_{x}{T}_{2}|}{2}\mathrm{.}$$

### Data availability

The datasets generated and analyzed during the current study are available from the corresponding author on reasonable request.
